# Polymorphism in the *Yersinia* LcrV Antigen Enables Immune Escape From the Protection Conferred by an LcrV-Secreting *Lactococcus Lactis* in a Pseudotuberculosis Mouse Model

**DOI:** 10.3389/fimmu.2019.01830

**Published:** 2019-08-02

**Authors:** Catherine Daniel, Amélie Dewitte, Sabine Poiret, Michaël Marceau, Michel Simonet, Laure Marceau, Guillaume Descombes, Denise Boutillier, Nadia Bennaceur, Sébastien Bontemps-Gallo, Nadine Lemaître, Florent Sebbane

**Affiliations:** Université de Lille, CNRS, Inserm, CHU Lille, Institut Pasteur de Lille, U1019 - UMR 8204 - Center for Infection and Immunity of Lille, Lille, France

**Keywords:** plague, *Yersinia pestis*, vaccine, LcrV, immune escape, polymorphism, probiotic, *Lactoccus lactis*

## Abstract

Yersinioses caused by *Yersinia pestis, Yersinia pseudotuberculosis*, and *Yersinia enterocolitica* are significant concerns in human and veterinary health. The link between virulence and the potent LcrV antigen has prompted the latter's selection as a major component of anti-*Yersinia* vaccines. Here, we report that (i) the group of *Yersinia* species encompassing *Y. pestis* and *Y. pseudotuberculosis* produces at least five different clades of LcrV and (ii) vaccination of mice with an LcrV-secreting *Lactococcus lactis* only protected against *Yersinia* strains producing the same LcrV clade as that of used for vaccination. By vaccinating with engineered LcrVs and challenging mice with strains producing either type of LcrV or a LcrV mutated for regions of interest, we highlight key polymorphic residues responsible for the absence of cross-protection. Our results show that an anti-LcrV-based vaccine should contain multiple LcrV clades if protection against the widest possible array of *Yersinia* strains is sought.

## Introduction

*Yersinia pestis, Yersinia pseudotuberculosis*, and *Yersinia enterocolitica* are bacterial pathogens in humans and animals (1). *Y. pestis* causes plague while *Y. enterocolitica* causes bowel disease and *Y. pseudotuberculosis* causes tuberculosis-like symptoms in animals or Far East scarlet-like fever if it infects humans. However, some patients with enteric yersiniosis also develop immunopathological complications, such as reactive polyarthritis and erythema nodosum (2, 3). Furthermore, fatal hematogenous spreading of *Y. enterocolitica* and *Y. pseudotuberculosis* is often reported in immunocompromised individuals or patients with iron overload (3–5). Although plague outbreaks are reported in both hemispheres, outbreaks of human enteric yersiniosis occur mostly in the northern hemisphere (i.e., northern Europe, the United States, Canada, Russia, and Japan) (6, 7). It is noteworthy that human enteric yersiniosis is among the five foodborne illnesses with the greatest economic burden (8). In addition to the diseases' impact on human health, all three yersinioses are associated with a major socio-economic burden because they cause recurrent, fatal epidemics among livestock and in zoological and wildlife parks (9–17). Yersinioses are thus considered to be significant human and veterinary health problems. Unsurprisingly, research geared toward the production of anti-yersiniosis vaccines is now underway.

Today's anti-*Yersinia* vaccine development programs are focused on the major virulence factor LcrV, also referred to as V antigen (18–20). LcrV is a polymorphic protein (21–24). Most amino-acid variations occur in the second half-part of the protein, within which a hypervariable region has been identified (25). The residues at positions 51 and 63 are known to be highly polymorphic (26). Indeed, LcrV antigens have been classified into different types, subtypes and variants on the basis of (i) the length of the hypervariable region, (ii) the polymorphisms at residues 51 and 63, and (iii) other subtle sequence differences (21, 23, 25, 26). Furthermore, there is debate as to whether immunization using a single V type, subtype or even variant confers cross-protection against challenge with a *Yersinia* strain producing another V type, subtype or variant (22, 24, 25, 27–30). For instance, passive immunization of mice with rabbit anti-sera raised against purified V antigen from *Y. pestis* protected against plague and pseudotuberculosis (27). Moreover, Motin et al. also showed that anti-V antigen (from *Y. pseudotuberculosis*) is effective in providing passive immunity against plague and pseudotuberculosis but clearly ineffective against an infection by *Y. enterocolitica* indicating that a potential problem with cross-protection may exist (27). Furthermore, passive immunization with rabbit anti-sera raised against purified V antigen from *Y. pestis* protected against *Y. pestis* strains in which *lcrV* had been exchanged with that of *Y. enterocolitica* (24). In contrast, we have reported that active immunization with *Lactococcus lactis* secreting the V antigen from *Y. pseudotuberculosis* confers protection against pseudotuberculosis but not against plague (28). Hence, the present study was designed to establish why there was an absence of cross-protection after active immunization with an LcrV-producing *L. lactis*.

## Materials and Methods

### Bacterial Strains, Plasmids, and Growth Conditions

We used *Y. pseudotuberculosis* strains (from our own collection and isogenic mutants generated in the present study), *L. lactis* strain MG1363, *Escherichia coli* SM10 λpir, the lactococcal pNZYR expression vector conferring resistance to chloramphenicol, and the pCVD442 suicide vector conferring resistance to ampicillin and sensitivity to sucrose. *Y. pseudotuberculosis, E. coli*, and *L. lactis* were, respectively, cultured in lysogeny broth at 28°C, lysogeny broth at 37°C and M17 medium supplemented with 0.5% glucose at 30°C. Growth media were supplemented with chloramphenicol (10 μg/ml) or ampicillin (100 μg/ml) when required. Custom pNZYR vectors (Eurogentec) containing the *lcrVs* of interest (translationally fused with the *L. lactis usp45* secretion signal sequence and under the control of the *Pusp45* promoter) were generated as previously described (28). Mutants of *Y. pseudotuberculosis* in which *lcrV* is mutated in the region of interest were generated by allelic exchange after mating *Y. pseudotuberculosis* with *E. coli* strain SM10 λpir harboring the pCVD442 plasmid containing an insert of interest as previously described (31, 32). Inserts of interest were obtained by overlapping PCR using the primer sets 5′-ACATGCATGCGTCATGGTTCTTCAGTTTTA-3′/5′-TATAAGAGTATGAGTTTTTCAGATTACCCAACGCCCCGGT-3′ and 5′-TTGGGTAATCTGAAAAACTCATACTCTTATAATAAAGATA-3′/5′-AAAAAGAGCTCCGTTGAGCATGGCGATAGTT-3′ and *Y. pestis* 195/P DNA template. Amplicons were digested with *Sph*I*/Sac*I endonucleases thanks to the restriction sites found in the primers (see underlined bases), purified then ligated to the *Sph*I/*Sac*I-restricted pCVD442 via standard T4 DNA ligase procedures. Mutations were verified by nucleotide sequencing. The *Y. pseudotuberculosis* strains producing the mutated LcrV were found to be fully virulent in mice using the same protocol as described below.

### Evaluation of the Efficacy of Immunization

Groups (*n* = 9–12) of 8-week-old, female BALB/c mice (Charles River) were immunized intranasally with sterile, phosphate-buffered saline (PBS) containing 10^9^ CFU of *L. lactis* producing (or not) the V antigen of interest, as previously described (28). Immunization was repeated on days 2, 22, 23, 43, and 44. One week after the final boost, serum was collected from animals and the anti-V titers were measured as previously described (28). Notably, microtiter well plates (Nunc-Immuno Plate) were coated with purified recombinant *Y. pestis* LcrV in PBS (pH 7.2) at 50 ng per well. Following overnight incubation, wells were blocked in PBS with 0.05% Tween 20, 3% bovine serum albumin (BSA). Blood samples were tested using 2 fold serial dilutions (from 1:50 and 1:2) in PBS with 0.05% Tween 20, 1% BSA. Specific antibody binding to LcrV was detected using horseradish peroxidase-conjugated rat anti-mouse IgG (BD Biosciences). The IgG antibody titer is given in terms of dilutions as described previously (28). Briefly, the OD450 was measured with an Elx800GUV automated microplate reader (Bio-Tek Instruments Inc., Vinooski, VT). End-point titers were calculated as the reciprocal of the dilution producing the same OD450 as three times the background using the KC4 program (Kineticalc for Windows, Bio-Tek Instruments). Lastly, 2 weeks after the final boost, mice were inoculated intravenously with 300 μl of PBS containing 10^3^ *Y. pseudotuberculosis* (a dose that we assessed to be above the LD50 for all the strains used prior challenge of immunized mice). The mice's survival was monitored daily for 3 weeks.

### Sequencing, Translation, and Phylogenetic Analysis

DNA from *Y. pseudotuberculosis* strains was purified using the Nucleospin kit (Macherey-Nagel) and then used to amplify *lcrV* by PCR using the primer sets 5′-TCACCGCGCAAAATTATTGC-3′; 5′-TTGTCTGCGATAAGCTCTT G-3′. Purified amplicons were sequenced with the primers used for amplification and the following primers: 5′-CCTAGCTTATTTTCTACCCG-3′; 5′-GAACCGGGGCGTTGGGTAATC-3′; 5′-GTTGGTTGTCATAATGACCGCC-3′; 5′-CTAACCAAGTCGTTGAGCGG-3′. Nucleotide sequences were translated using the ExPaSy Translate tool (http://web.expasy.org/translate/). Lastly, a phylogenic tree based on LcrV sequences was generated using the (http://phylogeny.lirmm.fr/phylo_cgi/index.cgi) web server (33).

### Evaluation of Plasmid Stability

The stability of the various expression vectors containing the *lcrV* gene of interest and conferring resistance to chloramphenicol in *L. lactis* MG1363 was checked by growing bacteria in M17 broth. After 10 subcultures (~100 generations) in the absence of any selective pressure, bacteria were plated on M17 agar plates with or without chloramphenicol, in order to count chloramphenicol-resistant and -sensitive CFUs. Furthermore, the secretion of LcrV from three randomly clones grown on M17 agar without chloramphenicol was determined by Western-blot analysis.

### Immunobloting and SDS-PAGE

For *Y. pseudotuberculosis*, overnight cultures of bacteria grown in LB at 28°C were diluted 1:20 into fresh LB containing 2.5 mM CaCl_2_. After 2 h incubation at 37°C, the optical density (OD) at 600 nm of the cultures was measured, then bacterial cells were collected after centrifugation at 4°C for 20 min and suspended in PBS such as each suspension of interest reaches an equivalent bacterial load. Bacterial suspensions were admixed with Laemmli buffer with β mercaptoethanol. Whole-cell lysates were separated by sodium dodecyl sulfate-polyacrylamide gel electrophoresis (SDS-PAGE). Notably, two gels were run at the same time. One gel was stained with Coomassie blue dye to control the loading and the other gel was used to electrophoretically transfer proteins to a nitrocellulose membrane. Immunoblot analyses were performed using polyclonal rabbit antibodies to *Y. pestis* LcrV (as primary antibody) (34) and an anti-rabbit IgG conjugated to horseradish peroxidase (as secondary antibody). Immunoreactive proteins were visualized and quantified by chemiluminescence using the LAS-3000 apparatus (Fujifilm) and the software Multi-Gauge (Fujifilm), respectively. The signal intensities reported here have a linear relationship with antigen loading (data not shown). For recombinant *L. lactis* secreting LcrV, cultures from an overnight growth at 37°C and with equivalent ODs were centrifuged and bacterial culture supernatants were filtered through 0.22 μm Millex HA membranes. Quantity of LcrV secreted by *L. lactis* was determined by chemiluminescence as described above for *Y. pseudotuberculosis*. It was further confirmed by SDS-PAGE and Coomassie blue staining using a culture supernatant prepared another a day than that of used for quantification by chemiluminescence.

### Statistical Analysis

The Mann-Whitney *U* test and the log-rank test were used to compare antibody titers and survival curves, respectively.

## Results

### *L. lactis* Secreting LcrV From *Y. pestis* Does Not Confer Protection Against *Y. pseudotuberculosis* Strain 2777

**Figure 1:**
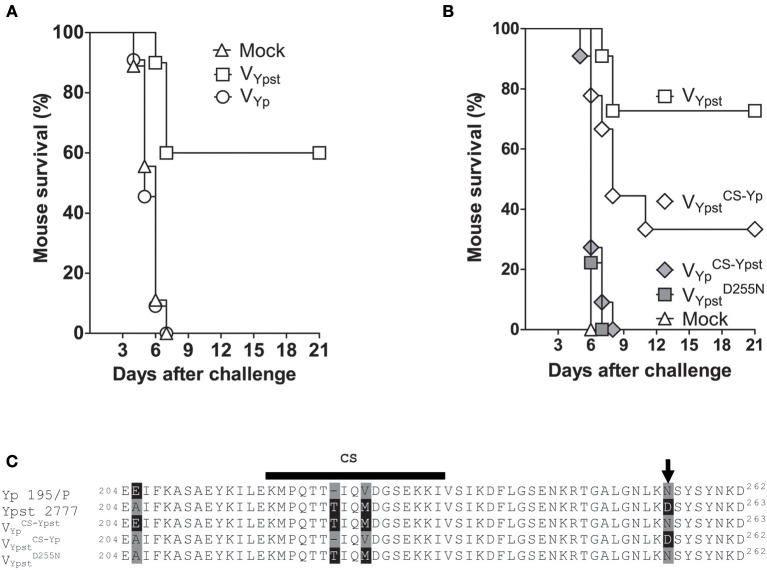
Subtle substitutions in the V antigen used for vaccination enable *Yersinia pseudotuberculosis* to escape an immune response against LcrV. **(A,C)** The survival rate of mice (*n* = 9–11) vaccinated intranasally with *L. lactis* alone (Mock), or *L. lactis* secreting the V antigen from *Y. pseudotuberculosis* strain 2777 (V_Ypst_), the V antigen from *Y. pestis* strain 195/P (V_Yp_), V_Ypst_ with a substitution Asp to Asn at the position 255 (${\text{V}}_{\text{Ypst}}{\;}^{\text{D255N}}$), or V_Ypst_ or V_Yp_ in which the “conformational segment” (CS) had been exchanged (${\text{V}}_{\text{Ypst}}{\;}^{\text{CS}-\text{Yp}}$ and ${\text{V}}_{\text{Yp}}{\;}^{\text{CS}-\text{Ypst}}$). Mice were significantly protected (*p* < 0.05 in a log-rank test) against *Y. pseudotuberculosis* strain 2777 only when they were vaccinated with V_Ypst_ or ${\text{V}}_{\text{Ypst}}{\;}^{\text{CS}-\text{Yp}}$ **(A,C)**. The difference in protection observed in mice vaccinated with V_Ypst_ and ${\text{V}}_{\text{Ypst}}{\;}^{\text{CS}-\text{Yp}}$ was not significant (*p* > 0.07 in a log-rank test). **(B)** Alignments of the protective regions of interest in LcrV proteins. The arrowhead indicates the residue at position 255.

Although the LcrV proteins from *Y. pestis* 195/P and *Y. pseudotuberculosis* strain 2777 share more than 97% sequence identity, we previously reported that mice immunized with *L. lactis* secreting the LcrV from *Y. pseudotuberculosis* strain 2777 were protected against pseudotuberculosis but not against plague (28). This surprising finding suggested that active immunization with LcrV from *Y. pestis* should be ineffective against *Y. pseudotuberculosis*. To test this hypothesis, we generated a strain of *L. lactis* that secreted LcrV from *Y. pestis* (V_Yp_) and then assessed whether this new strain afforded protection against *Y. pseudotuberculosis*. To this end, groups of mice were immunized intranasally with *L. lactis* secreting V_Yp_ or that does not synthetize any LcrV (control strain), according to the previously reported protocol (28). As an additional control, a group of mice was immunized with *L. lactis* secreting LcrV from *Y. pseudotuberculosis* (V_Ypst_). Two weeks after the end of the immunization protocol, mice were challenged intravenously with a lethal dose of *Y. pseudotuberculosis* strain 2777. The proportion of animals developing fatal pseudotuberculosis was similar in mice vaccinated with V_Yp_ and in unvaccinated mice (Figure 1A). This contrasted with the higher survival rate observed in animals vaccinated with V_Ypst_ (Figure 1A). The absence of cross protection was confirmed in an independent experiment.

### Vaccination With the LcrV From *Y. pseudotuberculosis* Strain 2777 With a Single Substitution at Position 255 Does Not Confer Protection Against *Y. pseudotuberculosis* Strain 2777

The sequences of the LcrV proteins from *Y. pseudotuberculosis* strain 2777 and *Y. pestis* strain 195/P used in the above immunization experiments differ in the region considered to contain a plague-protective epitope (27, 35–37). In particular, the *Y. pestis* LcrV region lacks a threonine and has three residue substitutions: E205A, V226M, and N255D (Figure 1B). It is noteworthy that the missing threonine and the substituted residue 226 are located within the 16-amino acid LcrV segment (residues 218–234), which is determinant for protein conformation and is referred to here as the “conformational segment” (CS) (27, 38). These residues are part of the immunodominant epitopes recognized by H-2d CD4 T cells and known be the major protective epitope (35, 39, 40). Furthermore, the Asn-255 in the *Y. pestis* LcrV is known to be a key residue for the binding of a plague-protective monoclonal antibody, since the substitution N255D greatly reduces the antibody's affinity for LcrV (41). Given that substitution of E205A may not impact protein conformation, we hypothesized that the residue substitutions in the conformational segment and/or at position 255 were responsible for the absence of cross-protective immunity observed in our experiments. To test this hypothesis, we generated *L. lactis* strains secreting a hybrid *Y. pestis*/*Y. pseudotuberculosis* LcrV protein in which the conformational segments had been exchanged (yielding ${\text{V}}_{\text{Ypst}}{\;}^{\text{CS}-\text{Yp}}$ and ${\text{V}}_{\text{Yp}}{\;}^{\text{CS}-\text{Ypst}}$) or with an Asp to Asn substitution at position 255 in V_Ypst_ (yielding ${\text{V}}_{\text{Ypst}}{\;}^{\text{D255N}}$) (Figure 1B). After immunization as described above, we assessed the degree of protection conferred against a lethal intravenous challenge of *Y. pseudotuberculosis* strain 2777 in the various groups of mice. All mice immunized with *L. lactis* alone or *L. lactis* secreting ${\text{V}}_{\text{Yp}}{\;}^{\text{CS}-\text{Ypst}}$ and ${\text{V}}_{\text{Ypst}}{\;}^{\text{D255N}}$ died, furthermore, the time courses of mortality were similar (*p* > 0.05) in the three groups (Figure 1C). Hence, ${\text{V}}_{\text{Yp}}{\;}^{\text{CS}-\text{Ypst}}$ and ${\text{V}}_{\text{Ypst}}{\;}^{\text{D255N}}$ did not confer any protection against pseudotuberculosis. In contrast, immunization with *L. lactis* secreting V_Ypst_ and ${\text{V}}_{\text{Ypst}}{\;}^{\text{CS}-\text{Yp}}$ afforded protection (Figure 1C). However, the survival rate was higher in mice immunized with V_Ypst_ than with ${\text{V}}_{\text{Ypst}}{\;}^{\text{CS}-\text{Yp}}$ (~75 and ~35%, respectively). Similar results were obtained in an independent experiment: absence of cross-protection with ${\text{V}}_{\text{Yp}}{\;}^{\text{CS}-\text{Ypst}}$ and V_Ypst_^D255N^ and, the survival rates of mice vaccinated with V_Ypst_ and ${\text{V}}_{\text{Ypst}}{\;}^{\text{CS}-\text{Yp}}$ were 50 vs. 20%, respectively. However, the difference in protection between mice vaccinated with V_Ypst_ and ${\text{V}}_{\text{Ypst}}{\;}^{\text{CS}-\text{Yp}}$ against pseudotuberculosis in both experiments was not statistically significant (*p* > 0.07 in a Gehan-Wilcoxon test). Taken as a whole, these data indicate that a single substitution at position 255 of LcrV seems to abolish the vaccine efficacy.

### Protection Against Pseudotuberculosis by Vaccination With LcrV Is Dependent on the *Y. pseudotuberculosis* LcrV Clade

**Figure 2:**
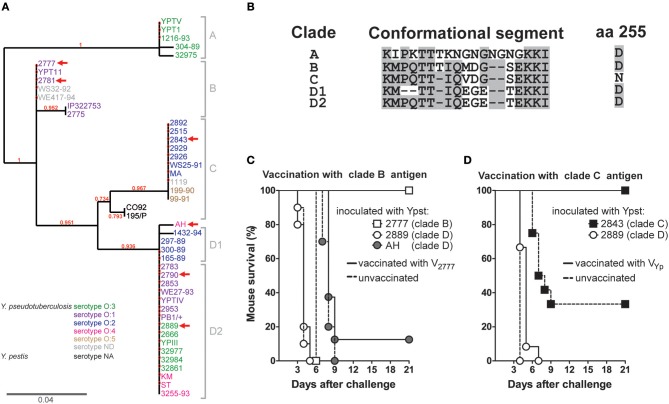
The different clades of V antigen and their respective degrees of protective immunity. **(A)** The phylogeny of the V antigen. The tree was generated from predicted amino-acid sequences of 43 strains of *Y. pseudotuberculosis* and 2 strains of *Y. pestis* (C092 and 195/P), using “One-Click” phylogenetic analysis software (http://phylogeny.lirmm.fr/phylo_cgi/index.cgi) (33). The bootstrap values are indicated for each branch. The scale bar indicates the amount of change. The length of a branch is proportional to the number of substitutions per site. Strains that belong to the same clade are indicated by the letter **(A–D)** shown on the right side of the tree. However, the clade D has been subdivided into two groups on the basis of the sequence of the CS, as shown in **(B)**. Red arrowheads indicate the V antigen and the strains used in the experiments are shown in panels **C,D** and in the Supplementary Figure 1. Lastly, the serotype of each strain is given; ND, not determined; NA, not applicable. The amino-acid sequences of all the strains used to generate the tree are given in the Supplementary Material. **(B)** Alignments of the CS and the residue at position 255 of LcrV from different clades. **(C,D)** The survival rate of mice (*n* = 9–10) vaccinated intranasally with *L. lactis* alone (Mock) or *L. lactis* secreting the V antigen from *Y. pseudotuberculosis* strain 2777 (V_2777_; clade B) or the V antigen from *Y. pestis* strain 195/P (V_Yp_; clade C), after challenge with strains 2777 (clade B), 2889, AH (both clade D) (C), 2843 (clade C), or 2889 (clade D) **(D)**. Mice were significantly protected (*p* < 0.05 in a log-rank test) against *Y. pseudotuberculosis* strains 2777 and 2843.

Although the above-mentioned difference in protection resulting from the exchange of the conformational segment (i.e., vaccination with ${\text{V}}_{\text{Ypst}}{\;}^{\text{CS}-\text{Yp}}$) was not statistically significant, we suspected that it was biologically significant. We thought that the difference in protection was due to the fact that the amino-acid sequences of the conformational segments in the *Y. pseudotuberculosis* 2777 and *Y. pestis* 195/P strains are almost identical (Figure 1B). Hence, we hypothesized that *L. lactis* secreting the LcrV from *Y. pseudotuberculosis* strain 2777 (hereafter referred to as V_B_) would not confer protection against strains producing a LcrV composed of (i) a conformational segment strongly differing from that of V_B_ and (ii) the same residue as V_B_ at position 255 (an Asp) since this latter seems important for immune escape. To test this hypothesis, we first sought to identify strains producing a V antigen that meet our criteria. To this end, we sequenced *lcrV* from our collection of 43 *Y. pseudotuberculosis* strains. Amino-acid sequence and phylogenetic analyses listed several strains of interest which cluster into five major clades (V_A_ to V_D2_) (Figure 2A). LcrV from each of the five clades had its own distinct conformational segment. The V_A_, V_B_, V_D1_ and V_D2_ antigens have an Asp at position 255, whereas the V_C_ antigen has an Asn (Figure 2B). Hence, we immunized mice with *L. lactis* secreting V_B_ and measured the animals' survival after inoculation with *Y. pseudotuberculosis* strain 2889, AH, and 2790 producing a V_D_ antigen and the strain 2777 producing the V_B_ antigen used for vaccination. As an additional and independent experiment, we monitored survival in mice immunized with *L. lactis* secreting V_B_ after challenging with the 2781 strain whose V antigen sequence is identical to that used for vaccination. Even though the vaccinated mice mounted a strong anti-V response (data not shown), the vaccination neither significantly decreased the mortality rate nor affected the kinetics of mortality in mice inoculated with strains 2889, AH, and 2790 (Figure 2C and Supplementary Figure 1A). In contrast, more than 90% of mice infected with the strain 2777 and 2781 survived (Figure 2C and Supplementary Figure 1B). This total absence of cross-protection between V_Ypst_ variants supports the hypothesis whereby the residues in the conformational domain influence protective immunity.

### LcrV From *Y. pestis* Confers Protection Against *Y. pseudotuberculosis* Strains Producing the Same LcrV Clade as *Y. pestis*

A phylogenetic analysis of *Y. pestis* strains CO92 and 195/P and our *Y. pseudotuberculosis* strains revealed that *Y. pseudotuberculosis* LcrV clade C was closely related to *Y. pestis* LcrV (Figure 2A). Further sequence analysis showed that LcrV proteins from this particular clade have the same conformational domain sequence and an Asn residue at position 255 as LcrV from *Y. pestis* (i.e., V_Yp_ = V_C_). Bearing in mind that the residues of the conformational segment and at position 255 might influence protective immunity (Figures 1A,C, 2C and Supplementary Figure 1), we predicted that LcrV from *Y. pestis* (i.e., V_C_) should protect against *Y. pseudotuberculosis* strains producing a V_C_ antigen but not another V clade. In line with our prediction, mice vaccinated with the V_C_ antigen were protected against pseudotuberculosis caused by the strain 2843 producing a V_C_ antigen but not against the strain 2889 which synthesizes a V_D_ antigen (see the red arrowheads in Figures 2A,D). Lastly, it is noteworthy to highlight that vaccination with V_C_ (V_Yp_) did not confer protection against a lethal challenge of *Y. pseudotuberculosis* strain 2777 (Figure 1A), which produces V_B_ (Figure 2A). In conclusion, our above and present data show that vaccination of mice with the LcrV antigen protects against *Yersinia* strains producing the same LcrV clade as that used for vaccination.

### Polymorphism in the LcrV Antigen Enables Immune Escape

**Figure 3:**
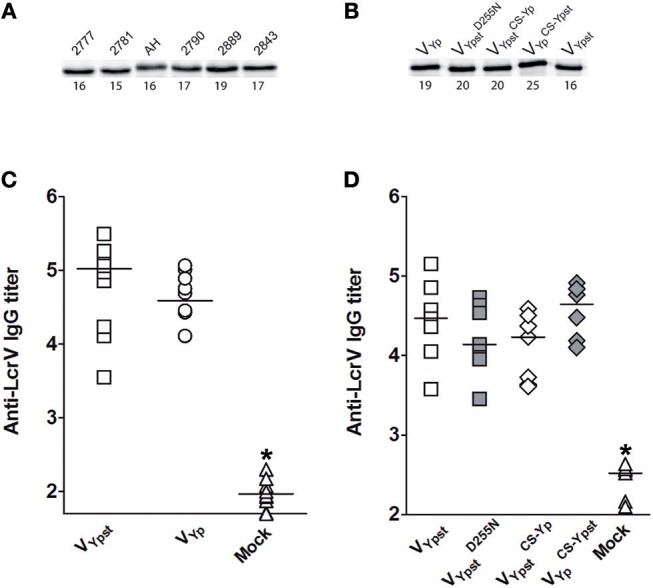
*Y. pseudotuberculosis* strains produce similar amounts of V antigen, and recombinant *L. lactis* strains secrete similar amounts of V antigen and induce similar anti-V IgG antibody titers in vaccinated mice. An immunoblot of **(A)** whole-cell lysate of *Y. pseudotuberculosis* strains and **(B)** culture supernatants from *L. lactis* secreting the V antigen from *Y. pestis* strain 195/P (V_Yp_), the V antigen from *Y. pseudotuberculosis* strain 2777 (V_Ypst_), V_Ypst_ with an Asp to Asn substitution at position 255 (${\text{V}}_{\text{Ypst}}{\;}^{\text{D255N}}$), or V_Ypst_ or V_Yp_ in which the “conformational segment” (CS) had been swapped ${\text{(V}}_{\text{Ypst}}{\;}^{\text{CS}-\text{Yp}}\text{ and }{\text{V}}_{\text{Yp}}{\;}^{\text{CS}-\text{Ypst}})$. The number beneath each band indicates a relative density value (expressed as a percentage), which was calculated by dividing the density of the band of interest by the sum of the density of all the bands shown on the blot (See Supplementary Figure 3 for raw data). **(C,D)** Antibody titers shown in the panels were measured using sera from the mice used in the experiments shown in Figures 1A,C, respectively. Horizontal lines indicate the mean titers. Regardless of the V antigen used for vaccination, the antibody titer against the V antigen of the mock group is significantly different than the antibody titers from mice vaccinated with a V antigen (^*^p < 0.003 a Mann-Whitney *U* test). The antibody titers measured in the various groups of mice did not differ significantly as a function of the V antigen used (*p* > 0.13 in a Mann-Whitney *U* test).

**Figure 4:**
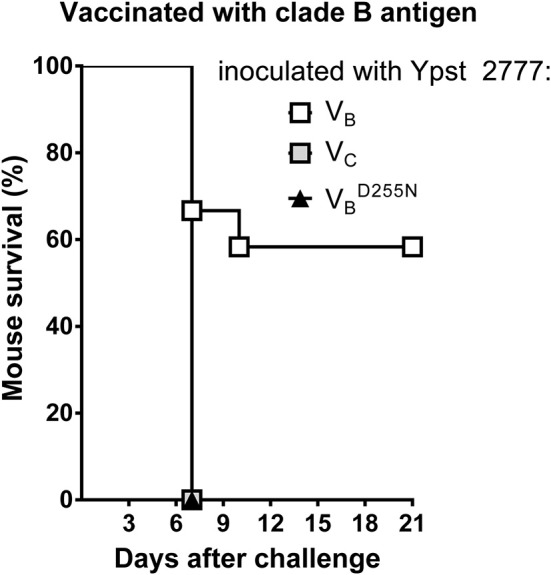
Subtle polymorphism in the *Yersinia* LcrV antigen enables immune escape from the protection conferred by LcrV. The survival rate of mice (*n* = 9–11) vaccinated intranasally with *L. lactis* secreting the V_B_ antigen from strain 2777 after challenge with strain 2777 or its isogenic mutants in which V_B_ had been mutated such as they synthetize a V_C_ antigen or a V_B_ antigen with an Asp to Asn substitution at position 255 ($\text{V}{\;}_{\text{B}}^{\text{D}255\text{N}}$). Mice were significantly protected against strain 2777 but not against its isogenic mutants (*p* < 0.05 in a log-rank test).

The absence of cross-protection reported in the different above sections does not appear to be dependent on the serotype of the *Y. pseudotuberculosis* strain used [Figure 2A (see the red arrowheads)]. It did not correlate either with the virulence of the strain used (Figure 2C). Lastly, it was not associated with the LcrV antigen production level between the different *Y. pseudotuberculosis* strains (Figure 3A). Nor does it appear to result from any instability of the lactococcal plasmid harboring the genetic construct of interest since fewer than 1% of *L. lactis* subcultured 10 times in the absence of selective pressure lost the plasmid. Lastly, it does not appear to result from an apparent difference in the amounts of V antigen secreted by the *L. lactis* strains (Figure 3B and Supplementary Figure 2). In agreement with these *in vitro* data, vaccinated mice had similar anti-V IgG titers regardless of the vaccine strain used (Figures 3C,D). In other words, our data support the idea that LcrV polymorphism enables escape from anti-V immunity. To confirm this conclusion, we generated isogenic mutants of *Y. pseutoduberculosis* strain 2777 in which V_B_ has been mutated such as the mutant strains produce either a V_c_ antigen or a V_B_ antigen with an Asp to Asn substitution at position 255 (i.e., $\text{V}{\;}_{\text{B}}^{\text{D}255\text{N}}$). Next, we compared the ability of the mutant and parental strains to escape immunity in mice vaccinated with the V_B_ antigen. Results showed that mice were protected against the parental strain but not against the isogenic mutants (Figure 4). In conclusion, LcrV antigen polymorphism enables immune escape and a single substitution at position 255 is sufficient for this escape.

## Discussion

Comparative sequence analysis of LcrV from the three pathogenic *Yersinia* species reveals two major types, three subtypes and several variants of the V antigen (21, 23, 25, 26). Here, we report there are at least five V variants produced by the group of *Yersinia* species encompassing *Y. pestis* and *Y. pseudotuberculosis* (Figure 2A). The results of former vaccination studies using purified recombinant V antigen produced in *E. coli* suggested that protection against all types of yersiniosis does not depend on the type, subtype or variant of V antigen used for vaccination (22, 41). Our previous results (28) and present data do not support the latter conclusion. Indeed, vaccination using LcrV-secreting *L. lactis* did not provide cross-protective immunity between different V antigen variants or even between different V subtypes and types (Figures 1, 2, 4 and Supplementary Figure 1). Furthermore, isogenic mutants producing LcrV mutated for residues of interest escape from the protection conferred by the original LcrV (Figure 4). However, our data are reminiscent (to some extent) of those recorded in passive immunization experiments. Cross-protection between different V types and subtypes was not observed unless a large amount of antibody was provided (22, 24, 25, 42, 43). Hence, it is conceivable that vaccination using recombinant *L. lactis* does not generate a sufficiently cross-protective antibody titer. Unfortunately, the fact that studies showing cross-protection after vaccination with recombinant V-antigen produced in *E. coli* did not report the antibody titers (22, 41) makes it impossible to evaluate the above-mentioned hypothesis at present. The discrepancy might also be due to differences in the cellular response generated by the vaccination using purified antigen vs. recombinant *L. lactis*.

The absence of cross-protection reported here indicates that polymorphism in the *Yersinia* LcrV antigen enables immune escape. LcrV is a polymorphic antigen within which the most effectively protective region encompasses residues 135 to 262 (37). The major protective epitope is thought to correspond to the highly polymorphic segment (residues 218–234) (22, 25, 27). However, this latter assumption had not been confirmed because the major epitope's residues had not been identified *per se* (22, 24). It is worth noting that immunization with a V antigen lacking the residues 240–271 provides almost no protection (44). In our present experiments, we used (i) *L. lactis* strains secreting a V antigen in which the hypervariable segment had been exchanged and (ii) *Y. pseudotuberculosis* strains producing a V antigen whose hypervariable segment differed significantly from that used for vaccination. Our results suggest that polymorphism in the hypervariable segment enables escape from an active immune response against LcrV (Figures 1, 2). One could consider that the absence of cross-protection reported here reflects factors that differ from one strain to another. Our previous (28) and present work show that vaccination with V_B_ antigen protects against strains producing V_B_ but not V_C_ or V_D_ antigen [Figure 2C, Supplementary Figure 1, and (28)] and vaccination with V_C_ antigen protects against strains producing Vc but not V_B_ or V_D_ antigen (Figures 1A, 2D). Hence, if the absence of cross-protection reflects factors that differ from one strain to another, our data would indicate that strains producing a V_B_, V_C_ and V_D_ antigen each produce a distinct, very efficient factor that enables escape from immunity directed against a V-antigen from a clade other than their own (Figures 1A,C, 2C,D and Supplementary Figure 1). However, the production of several (at least three) distinct, efficient factors for immune evasion is unlikely. Nonetheless, we acknowledge that vaccination assays using isogenic mutant strains will definitively end such a debate. However, regardless of the result of such a study, our data indicate that a V-based vaccine should be composed of several V antigens if it is to protect the host against a wide array of *Yersinia* strains or at least *Y. pseudotuberculosis* strains.

Although the role of polymorphism in the conformational segment in immune escape remains to be definitively proven, there is no doubt that residue at position 255 plays a critical role in the immune escape. Indeed, a single mutation at position 255 in the V antigen used for vaccination abolishes the efficacy of the vaccine and the same mutation in *Y. pseudotuberculosis* unables the mutant to escape from protection conferred by the original antigen (Figures 1C, 4). Interestingly, the three-dimensional structure of LcrV reveals that residues 218–234 and 255 are close to each other in space (38) and so might form a conformational epitope. However, the residues might also be part of two distinct epitopes that lead to the generation of cooperative antibodies. Consistent with the latter idea, a substitution at residue 255 annihilates the binding of the protective plague monoclonal antibody (mAb) 7.3 [which recognizes an epitope that does not contain the hypervariable segment (41)].

We previously reported that mucosal vaccination of mice with *L. lactis* secreting LcrV induced cell- and antibody-mediated protective immunity against *Y. pseudotuberculosis* infection in the mouse and the protection is long-lasting. We further reported that activated CD4^+^ T lymphocytes are necessary for vaccine-induced protection, whereas CD8^+^ T cells may have a moderate role. Several LcrV epitopes recognized by CD4 T-cell have been described in the literature for *Y. pestis* (39, 40, 45–47). Interestingly, residue at position 255 is the last residue of a strong T-cell epitope (GSENKRTGALGNLKN) for BALB/c mice (46, 47). It is unknown whether the last N residue of this epitope is important for vaccination using peptides. However, if it is important, our data suggest that vaccination should be performed using a mixture of peptides containing different residues at the end of the epitope. Moreover, further work should be performed to support this hypothesis because previous authors used linear peptides in their studies whereas we used the whole LcrV protein.

Although an anti-V humoral response is thought to be involved in protection, it is considered that the antiserum's ability to neutralize *Yersinia*-mediated toxicity against macrophages is more strongly correlated with vaccine efficacy than the anti-V antibody titer (48). In this context, the titer of native antibodies that bind the epitopes recognized experimentally by the monoclonal antibodies mAb7.3 and mAb-BA5 might be correlated with the degree of protection, since both mAb7.3 and mAb-BA5 protect against plague by neutralizing *Y. pestis*-mediated toxicity against host cells (22, 24). However, anti-V serum's ability to compete with mAb7.3 in binding to LcrV does not necessarily correlate with protection (49, 50)—suggesting that one should quantify the titer of antibodies that bind to mAb-BA5's epitope (rather than mAB7.3's epitope). Our data do not support the latter hypothesis, since mAb-BA5 recognizes an epitope (encompassing residues 196–224) that did not appear to contribute to protection in our experiments. In fact, our data suggest that only the titer of antibodies that recognize polymorphic epitopes comprising the hypervariable region and/or residue 255 is correlated with vaccine efficacy (Figure 1C). In other words, an antiserum's ability to neutralize *Yersinia*-mediated toxicity against macrophages may only be correlated with protection against a specific V variant. Unfortunately, setting up a vaccine efficacy assay based on macrophage toxicity is not trivial, considering that this methodology is based on regions whose residue composition (i) varies from one strain to another within the same species and (ii) may have changed during the evolution of the targeted strain.

The above conclusion contrasts with the observed correlation between anti-V titers on one hand and protection on the other (and even cross-protection between V antigen types) (22, 43, 51, 52). This discrepancy could be explained by an antibody titer threshold model in which a vaccine‘s protective/cross-protective efficacy is only correlated with the antibody titer when the latter is over a threshold value. Below this value, only the serum's ability to inhibit *Yersinia*-mediated toxicity against macrophages would be a marker of protection. However, data from this assay would probably be highly relevant for a targeted V variant (as mentioned above).

Lastly, our present data (in combination with previous results) strongly suggest that V-based vaccines using purified antigens or recombinant strains (such as *L. lactis*) as the antigen vehicle should be composed of multiple V antigens. The supporting evidence is as follows: (i) vaccination in humans using one LcrV variant may not necessarily induce the antibody titer threshold conferring cross-protection, since there is a difference in anti-V titers of several orders of magnitude between volunteers enrolled in clinical trial (53) and patients having experienced yersiniosis (54), (ii) only the most protective antibodies (which, according to our data, might be those recognizing polymorphic epitopes) persist over time (53), (iii) during the course of bacterial evolution, amino-acid substitutions may have occurred within the polymorphic, protective epitope, and (iv) the current assays thought to predict protection may be too restrictive (for the reasons described above).

In conclusion, we were able to identify at least one key residue composing the major protective epitopes in V antigen. Our present results indicate that a V-based vaccine should be composed of several V antigens if it is to protect the host against a wide array of *Yersinia* strains. However, this type of vaccine may fail to protect the host against strains producing a newly evolved V antigen. Consistently, the data further suggest that a vaccine efficacy assay other than that based on anti-V titer measurement and bacterial-mediated toxicity against macrophages must now be developed. Our general conclusion is based (i) on our previous study in which we reported that V antigen from *Y. pseudotuberculosis* does not confer protection against bubonic plague (28), and the present study using a pseudotuberculosis infection model and mice vaccinated with *L. lactis* secreting V antigens from *Y. pseudotuberculosis* and *Y. pestis*. Further work using *Y. pestis* and *Y. enterocolitica* with their respective LcrVs vaccine but also different animal models shall be done to fulfill our conclusions.

## Data Availability

The raw data supporting the conclusions of this manuscript will be made available by the authors, without undue reservation, to any qualified researcher.

## Ethics Statement

The animal experiments were carried out in accordance with the European directive 2010/63/EU, the French Decree No. 2013-118. All animal work was done according to protocols approved by the Ethics Committee in Animal Experimentation No.75 and the French Ministry of Higher Education, Research and Innovation.

## Author Contributions

CD and FS: conceived and designed the experiments, analyzed the data, and wrote the paper. CD, AD, SP, MM, MS, LM, GD, DB, NB, SB-G, and NL: performed the experiments.

### Conflict of Interest Statement

The authors declare that the research was conducted in the absence of any commercial or financial relationships that could be construed as a potential conflict of interest.
