# Supplementary material for: Polymorphism in the Yersinia LcrV Antigen Enables Immune Escape From the Protection Conferred by an LcrV-Secreting Lactococcus Lactis in a Pseudotuberculosis Mouse Model
Source: Front Immunol. 2019 Aug 2;10:1830. doi: 10.3389/fimmu.2019.01830 (PMC6688116; doi:10.3389/fimmu.2019.01830)
Supplement: Supplementary file 1 [file Data_Sheet_1.pdf]

Daniel_et_al_Supplementary_Figure_1

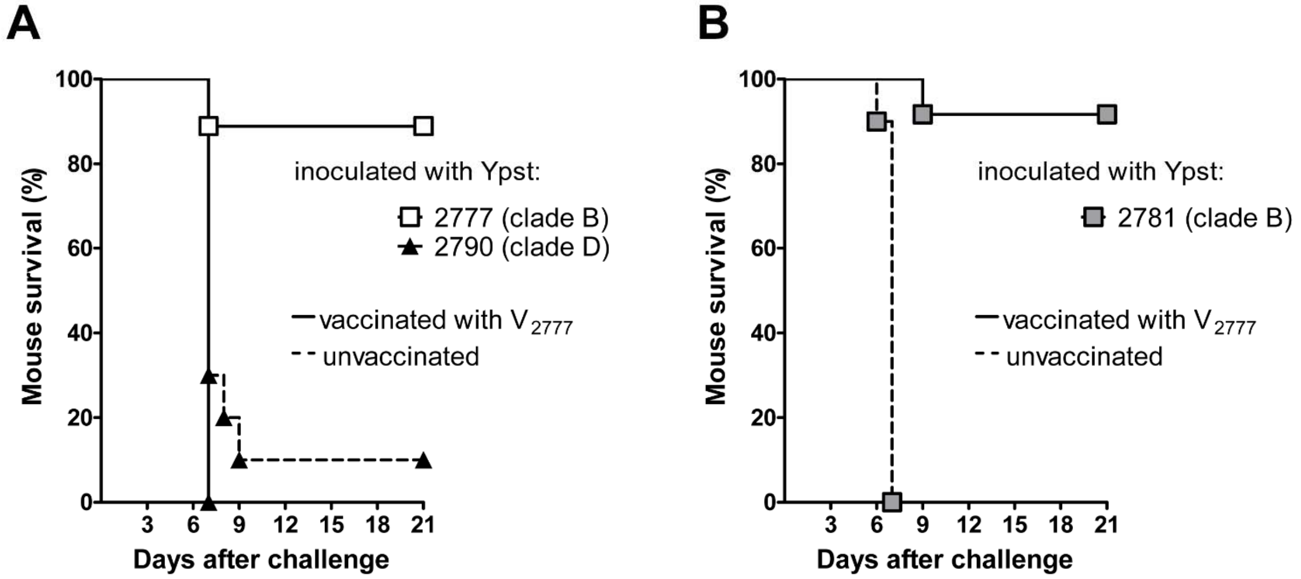

Supplementary Figure 1. *L. lactis* secreting clade B V antigen affords protection against *Y. pseudotuberculosis* strain producing clade B but not clade D V antigen. (A) Survival rate of mice (n=9 to 10) vaccinated intranasally with *L. lactis* alone or *L. lactis* secreting the V antigen from *Y. pseudotuberculosis* strain 2777 (V_{2777}; clade B) after challenge with strains 2777 (clade B) or 2790 (clade D). (B) Survival rate of mice (n=10 to 12) vaccinated intranasally with *L. lactis* alone or *L. lactis* secreting the V antigen from *Y. pseudotuberculosis* strain 2777 (V_{2777}; clade B) after challenge with strains 2781 (clade B).

Supplementary_Figure 2_Daniel_et_al

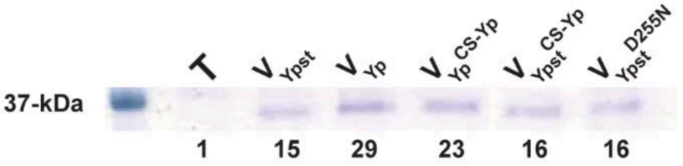

Supplementary Figure 2. Recombinant *L. lactis* strains secrete similar amounts of V antigen. A SDS-PAGE (stained with coomassie blue) of culture supernatants from *L. lactis* secreting the V antigen from *Y. pestis* strain 195/P (V_{Yp}), the V antigen from *Y. pseudotuberculosis* strain 2777 (V_{Ypst}), V_{Ypst} with an Asp to Asn substitution at position 255 (V_{ypst}^{D255N}), or V_{Ypst} or V_{Yp} in which the "conformational segment" (CS) had been swapped ($V_{ypst}^{CS\text{-}Yp}$ and $V_{yp}^{CS\text{-}Ypst}$) or harboring the empty pNZYR vector (T).

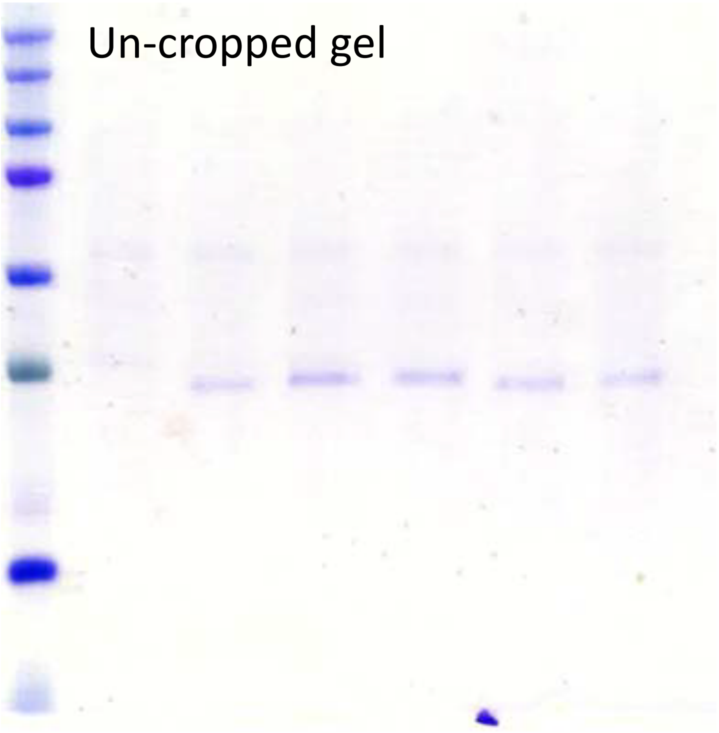

Supplementary_Figure 3_Daniel_et_al

A

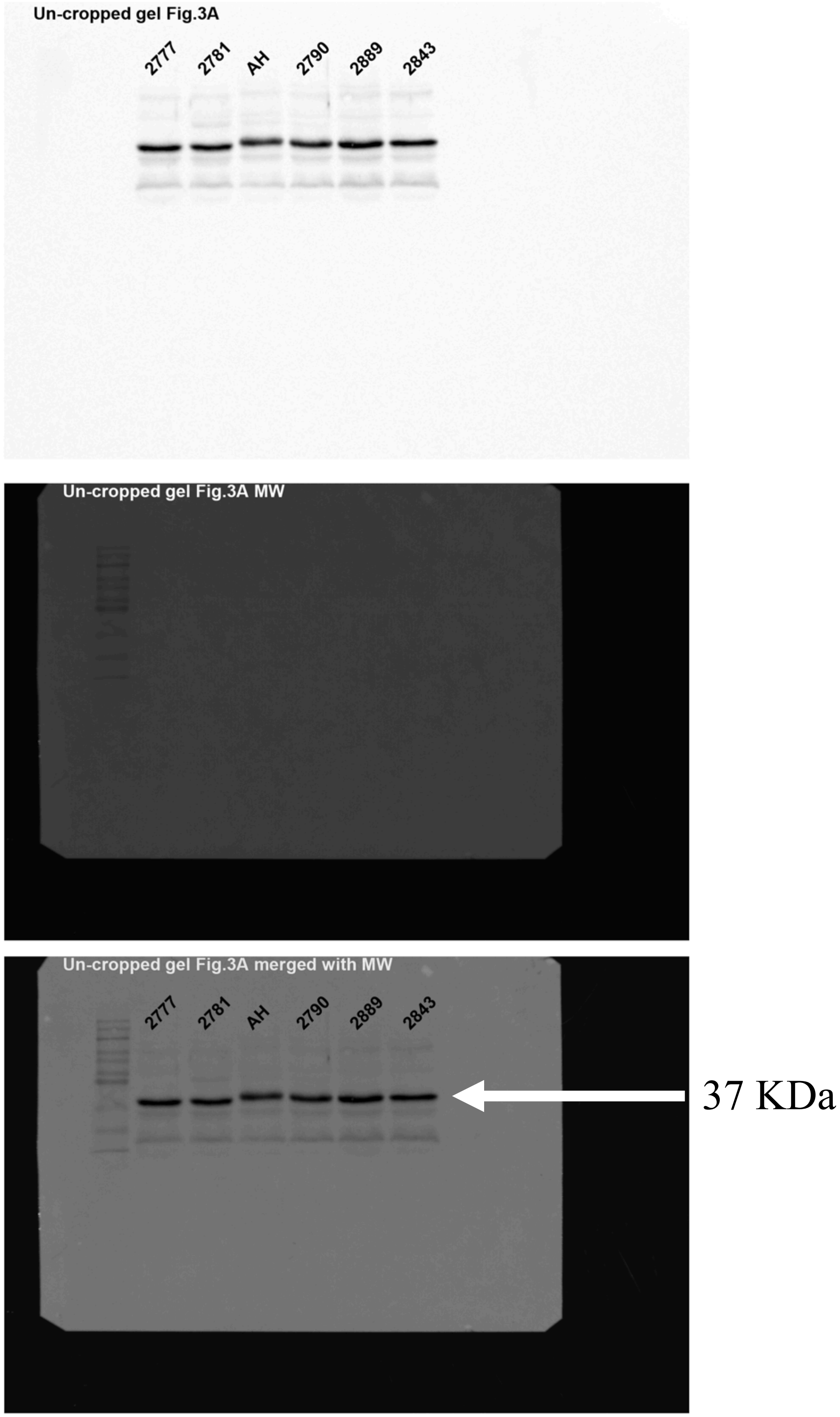

Supplementary Figure 3A. Uncropped gel from the immunoblot shown in Figure 3A. The immunoblot has been done on whole-cell lysates of different *Y. pseudotuberculosis* strains which produce similar amounts of V antigen. The arrow represents the LcrV antigen of 37 kDa. MW: molecular weight.

B

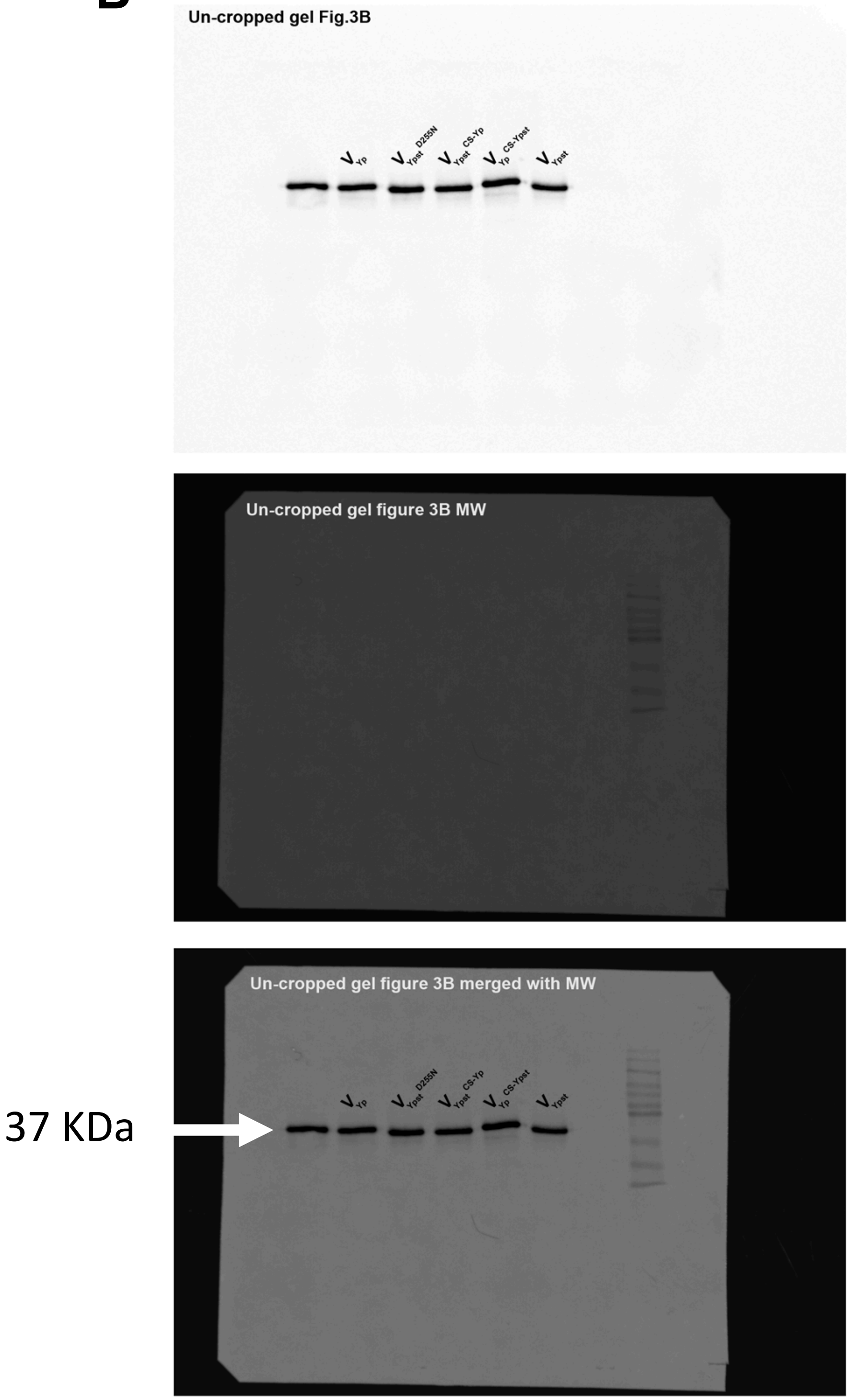

Supplementary Figure 3B. Uncropped gel from the immunoblot shown in Figure 3B. The immunoblot has been done on culture supernatants from recombinant *L. lactis* strains which secrete similar amounts of V antigen: *L. lactis* secreting the V antigen from *Y. pestis* strain 195/P (V_{Yp}), the Vantigen from *Y. pseudotuberculosis* strain 2777 (V_{Ypst}), V_{Ypst} with an Asp to Asn substitution at position 255 (V_{Ypst}^{D255N}), or V_{Ypst} or V_{Yp} in which the "conformational segment" (CS) had been swapped ($V_{Ypst}^{CS\text{-}Yp}$ and $V_{Yp}^{CS\text{-}Ypst}$). An extra band has been shown which represents LcrV from the culture supernatant of strain *Y. pseudotuberculosis* 2790. The arrow represents the recombinant LcrV antigen of 37 kDa
