# Supplementary material for: Polymorphism in the Yersinia LcrV Antigen Enables Immune Escape From the Protection Conferred by an LcrV-Secreting Lactococcus Lactis in a Pseudotuberculosis Mouse Model
Source: Front Immunol. 2019 Aug 2;10:1830. doi: 10.3389/fimmu.2019.01830 (PMC6688116; doi:10.3389/fimmu.2019.01830)
Supplement: Supplementary file 2 [file Data_Sheet_2.PDF]

Amino-acid sequences of all the strains used to generate the tree shown in Figure 2A.

GROUPE A

>YPTV
MIRAYEQNPQHFIEDLEKVRVEQLTGHGSSVLEELVQLVKDKNIDISIKYDPRKDSESFANRVITNDIELPKKILAYFLPEDAILKGGHYDNQL
QNGIKRVKEFLESSPNTQWELRAFMAVIHFSLTADRIDDDILKVIVDSMNHHGDARSKLREELAELTAELKIYSVIQAEINKHLSNSGTINISD
GSIDLMNKTLYGYTDEAIFKASAEYKILEKIPKTTTKNGNGNGNGNGNGNGNGNGNGNGKKIVSIKDFLGSENKRTGALGNLKDSYSYNKDNNE
LSHFATTCSDKSRPLNDLVSQKTTQLSDITSRFNSAIEALNRFIQKYDSVMQRLLDDTSAK

>YPT1
MIRAYEQNPQHFIEDLEKVRVEQLTGHGSSVLEELVQLVKDKNIDISIKYDPRKDSESFANRVITNDIELPKKILAYFLPEDAILKGGHYDNQL
QNGIKRVKEFLESSPNTQWELRAFMAVIHFSLTADRIDDDILKVIVDSMNHHGDARSKLREELAELTAELKIYSVIQAEINKHLSNSGTINISD
GSIDLMNKTLYGYTDEAIFKASAEYKILEKIPKTTTKNGNGNGNGKKIVSIKDFLGSENKRTGALGNLKDSYSYNKDNNELSHFATTCSDKSRP
LNDLVSQKTTQLSDITSRFNSAIEALNRFIQKYDSVMQRLLDDTSAR

>1216-93
MIRAYEQNPQHFIEDLEKVRVEQLTGHGSSVLEELVQLVKDKNIDISIKYDPRKDSESFANRVITNDIELPKKILAYFLPEDAILKGGHYDNQL
QNGIKRVKEFLESSPNTQWELRAFMAVIHFSLTADRIDDDILKVIVDSMNHHGDARSKLREELAELTAELKIYSVIQAEINKHLSNSGTINISD
GSIDLMNKTLYGYTDEAIFKASAEYKILEKIPKTTTKNGNGNGNGNGKKIVSIKDFLGSENKRTGALGNLKDSYSYNKDNNELSHFATTCSDKS
RPLNDLVSQKTTQLSDITSRFNSAIEALNRFIQKYDSVMQRLLDDTSAR

>304-89
MIRAYEQNPQHFIEDLEKVRVEQLTGHGSSVLEELVQLVKDKNIDISIKYDPRKDSESFANRVITNDIELPKKILAYFLPEDAILKGGHYDNQL
QNGIKRVKEFLESSPNTQWELRAFMAVIHFSLTADRIDDDILKVIVDSMNHHGDARSKLREELAELTAELKIYSVIQAEINKHLSNSDTINISD
GSIDLMNKTLYGYTDEAIFKASAEYKILEKIPKTTTKNGNGNGNGNGNGNGNGNGNGKKIVSIKDFLGSENKRTGALGNLKDSYSYNKDNNELS
HFATTCSDKSRPLNDLVSQKTTQLSDITSRFNSAIEALNRFIQKYDSVMQRLLDDTSAR

>32975
MIRAYEQNPQHFIEDLEKVRVEQLTGHGSSVLEELVQLVKDKNIDISIKYDPRKDSESFANRVITNDIELPKKILAYFLPEDAILKGGHYDNQL
QNGIKRVKEFLESSPNTQWELRAFMAVIHFSLTADRIDDDILKVIVDSMNHHGDARSKLREELAELTAELKIYSVIQAEINKHLSNSGTINISD
GSIELMNKTLYGYTDEAIFKASAEYKILEKISKTTTKNGNGNGNGNGNGNGNGNGNGNGNGKKIVSIKDFLGSENKRTGALGNLKDSYSYNKDN
NELSHFATTCSDKSRPLNDLVSQKTTQLSDITSRFNSAIEALNRFIQKYDSVMQRLLDDTSAR

GROUPE B

>2777
MIRAYEQNPQHFIEDLEKVRVEQLTGHGSSVLEELVQLVKDKNIDISIKYDPRKDSEFFANRVITDDIELLKKILAYFLPEDAILKGGHYDNQL
QNGIKRVKEFLESSPNTQWELRAFMAVIHFSLTADRIDDDILKVIVDSMNHHGDARSKLREELAELTAELKIYSVIQAEINKHLSSSGTINIHD
KSINLMDKNLYGYTDEAIFKASAEYKILEKMPQTTTIQMDGSEKKIVSIKDFLGSENKRTGALGNLKDSYSYNKDNNELSHFATTCSDKSRPLN
DLVSQKTTQLSDITSRFNSAIEALNRFIQKYDSVMQRLLDDTSAR

>YPT11
MIRAYEQNPQHFIEDLEKVRVEQLTGHGSSVLEELVQLVKDKNIDISIKYDPRKDSEFFANRVITDDIELLKKILAYFLPEDAILKGGHYDNQL
QNGIKRVKEFLESSPNTQWELRAFMAVIHFSLTADRIDDDILKVIVDSMNHHGDARSKLREELAELTAELKIYSVIQAEINKHLSSSGTINIHD
KSINLMDKNLYGYTDEAIFKASAEYKILEKMPQTTTIQMDGSEKKIVSIKDFLGSENKRTGALGNLKDSYSYNKDNNELSHFATTCSDKSRPLN
DLVSQKTTQLSDITSRFNSAIEALNRFIQKYDSVMQRLLDDTSAR

>2781
MIRAYEQNPQHFIEDLEKVRVEQLTGHGSSVLEELVQLVKDKNIDISIKYDPRKDSEFFANRVITDDIELLKKILAYFLPEDAILKGGHYDNQL
QNGIKRVKEFLESSPNTQWELRAFMAVIHFSLTADRIDDDILKVIVDSMNHHGDARSKLREELAELTAELKIYSVIQAEINKHLSSSGTINIHD
KSINLMDKNLYGYTDEAIFKASAEYKILEKMPQTTTIQMDGSEKKIVSIKDFLGSENKRTGALGNLKDSYSYNKDNNELSHFATTCSDKSRPLN
DLVSQKTTQLSDITSRFNSAIEALNRFIQKYDSVMQRLLDDTSAR

>WS32-92
MIRAYEQNPQHFIEDLEKVRVEQLTGHGSSVLEELVQLVKDKNIDISIKYDPRKDSEFFANRVITDDIELLKKILAYFLPEDAILKGGHYDNQL
QNGIKRVKEFLESSPNTQWELRAFMAVIHFSLTADRIDDDILKVIVDSMNHHGDARSKLREELAELTAELKIYSVIQAEINKHLSSSGTINIHD
KSINLMDKNLYGYTDEAIFKASAEYKILEKMPQTTTIQMDGSEKKIVSIKDFLGSENKRTGALGNLKDSYSYNKDNNELSHFATTCSDKSRPLN
DLVSQKTTQLSDITSRFNSAIEALNRFIQKYDSVMQRLLDDTSAR

>WE417-94
MIRAYEQNPQHFIEDLEKVRVEQLTGHGSSVLEELVQLVKDKNIDISIKYDPRKDSEFFANRVITDDIELLKKILAYFLPEDAILKGGHYDNQL
QNGIKRVKEFLESSPNTQWELRAFMAVIHFSLTADRIDDDILKVIVDSMNHHGDARSKLREELAELTAELKIYSVIQAEINKHLSSSGTINIHD
KSINLMDKNLYGYTDEAIFKASAEYKILEKMPQTTTIQMDGSEKKIVSIKDFLGSENKRTGALGNLKDSYSYNKDNNELSHFATTCSDKSRPLN
DLVSQKTTQLSDITSRFNSAIEALNRFIQKYDSVMQRLLDDTSAR

>IP322753
MIRAYEQNPQHFIEDLEKVRVEQLTGHGSSVLEELVQLVKDKNIDISIKYDPRKDSEFFANRVITGDIELLKKILAYFLPEDAILKGGHYDNQL
QNGIKRVKEFLESSPNTQWELRAFMAVIHFSLTADRIDDDILKVIVDSMNHHGDARSKLREELAELTAELKIYSVIQAEINKHLSSSGTINIHD
KSINLMDKNLYGYTDEAIFKASAEYKILEKMPQTTTIQMDGSEKKIVSIKGFLGSENKRTGALGNLKDSYSYNKDNNELSHFATTCSDKSRPLN
DLVSQKTTQLSDITSRFNSAIEALNRFIQKYDSVMQRLLDDTSAR

>2775
MIRAYEQNPQHFIEDLEKVRVEQLTGHGSSVLEELVQLVKDKNIDISIKYDPRKDSEFFANRVITGDIELLKKILAYFLPEDAILKGGHYDNQL
QNGIKRVKEFLESSPNTQWELRAFMAVIHFSLTADRIDDDILKVIVDSMNHHGDARSKLREELAELTAELKIYSVIQAEINKHLSSSGTINIHD
KSINLMDKNLYGYTDEAIFKASAEYKILEKMPQTTTIQMDGSEKKIVSIKGFLGSENKRTGALGNLKDSYSYNKDNNELSHFATTCSDKSRPLN
DLVSQKTTQLSDITSRFNSAIEALNRFIQKYDSVMQRLLDDTSAR

GROUPE C

>2892
MIRAYEQNPQHFIEDLEKVRVEQLTGHGSSVLEELVQLVKDKNIDISIKYDPRKDSEVFANRVITDDIELFKKILAYFLPEDAILKGGHYDNQL
QNGIKRVKEFLESSPNTQWELRAFMAVIHFSLTADRIDDDILKVIVDSMNHHGDARSKLREELAELTAELKIYSVIQAEINKHLSSSGTINIHD
ESINLMDKNLYGYTDDDIFKASAEYKILEKMPQTTIQVDGSEKKIVSIKDFLGSENKRTGALGNLKNSYSYNKDNNELSHFATTCSDKSRPLND
LVSQKTTQLSDITSRFNSAIEALNRFIQKYDSVMQRLLDDTSGK

>2515
MIRAYEQNPQHFIEDLEKVRVEQLTGHGSSVLEELVQLVKDKNIDISIKYDPRKDSEVFANRVITDDIELFKKILAYFLPEDAILKGGHYDNQL
QNGIKRVKEFLESSPNTQWELRAFMAVIHFSLTADRIDDDILKVIVDSMNHHGDARSKLREELAELTAELKIYSVIQAEINKHLSSSGTINIHD
ESINLMDKNLYGYTDDDIFKASAEYKILEKMPQTTIQVDGSEKKIVSIKDFLGSENKRTGALGNLKNSYSYNKDNNELSHFATTCSDKSRPLND
LVSQKTTQLSDITSRFNSAIEALNRFIQKYDSVMQRLLDDTSGK

>2843
MIRAYEQNPQHFIEDLEKVRVEQLTGHGSSVLEELVQLVKDKNIDISIKYDPRKDSEVFANRVITDDIELFKKILAYFLPEDAILKGGHYDNQL
QNGIKRVKEFLESSPNTQWELRAFMAVIHFSLTADRIDDDILKVIVDSMNHHGDARSKLREELAELTAELKIYSVIQAEINKHLSSSGTINIHD
ESINLMDKNLYGYTDDDIFKASAEYKILEKMPQTTIQVDGSEKKIVSIKDFLGSENKRTGALGNLKNSYSYNKDNNELSHFATTCSDKSRPLND
LVSQKTTQLSDITSRFNSAIEALNRFIQKYDSVMQRLLDDTSGK

>2929
MIRAYEQNPQHFIEDLEKVRVEQLTGHGSSVLEELVQLVKDKNIDISIKYDPRKDSEVFANRVITDDIELFKKILAYFLPEDAILKGGHYDNQL
QNGIKRVKEFLESSPNTQWELRAFMAVIHFSLTADRIDDDILKVIVDSMNHHGDARSKLREELAELTAELKIYSVIQAEINKHLSSSGTINIHD
ESINLMDKNLYGYTDDDIFKASAEYKILEKMPQTTIQVDGSEKKIVSIKDFLGSENKRTGALGNLKNSYSYNKDNNELSHFATTCSDKSRPLND
LVSQKTTQLSDITSRFNSAIEALNRFIQKYDSVMQRLLDDTSGK

>2926
MIRAYEQNPQHFIEDLEKVRVEQLTGHGSSVLEELVQLVKDKNIDISIKYDPRKDSEVFANRVITDDIELFKKILAYFLPEDAILKGGHYDNQL
QNGIKRVKEFLESSPNTQWELRAFMAVIHFSLTADRIDDDILKVIVDSMNHHGDARSKLREELAELTAELKIYSVIQAEINKHLSSSGTINIHD
ESINLMDKNLYGYTDDDIFKASAEYKILEKMPQTTIQVDGSEKKIVSIKDFLGSENKRTGALGNLKNSYSYNKDNNELSHFATTCSDKSRPLND
LVSQKTTQLSDITSRFNSAIEALNRFIQKYDSVMQRLLDDTSGK

>WS25-91
MIRAYEQNPQHFIEDLEKVRVEQLTGHGSSVLEELVQLVKDKNIDISIKYDPRKDSEVFANRVITDDIELFKKILAYFLPEDAILKGGHYDNQL
QNGIKRVKEFLESSPNTQWELRAFMAVIHFSLTADRIDDDILKVIVDSMNHHGDARSKLREELAELTAELKIYSVIQAEINKHLSSSGTINIHD
ESINLMDKNLYGYTDDDIFKASAEYKILEKMPQTTIQVDGSEKKIVSIKDFLGSENKRTGALGNLKNSYSYNKDNNELSHFATTCSDKSRPLND
LVSQKTTQLSDITSRFNSAIEALNRFIQKYDSVMQRLLDDTSGK

>MA
MIRAYEQNPQHFIEDLEKVRVEQLTGHGSSVLEELVQLVKDKNIDISIKYDPRKDSEVFANRVITDDIELFKKILAYFLPEDAILKGGHYDNQL
QNGIKRVKEFLESSPNTQWELRAFMAVIHFSLTADRIDDDILKVIVDSMNHHGDARSKLREELAELTAELKIYSVIQAEINKHLSSSGTINIHD
ESINLMDKNLYGYTDDDIFKASAEYKILEKMPQTTIQVDGSEKKIVSIKDFLGSENKRTGALGNLKNSYSYNKDNNELSHFATTCSDKSRPLND
LVSQKTTQLSDITSRFNSAIEALNRFIQKYDSVMQRLLDDTSGK

>1119
MIRAYEQNPQHFIEDLEKVRVEQLTGHGSSVLEELVQLVKDKNIDISIKYDPRKDSEVFANRVITDDIELFKKILAYFLPEDAILKGGHYDNQL
QNGIKRVKEFLESSPNTQWELRAFMAVIHFSLTADRIDDDILKVIVDSMNHHGDARSKLREELAELTAELKIYSVIQAEINKHLSSSGTINIHD
ESINLMDKNLYGYTDDDIFKASAEYKILEKMPQTTIQVDGSEKKIVSIKDFLGSENKRTGALGNLKNSYSYNKDNNELSHFATTCSDKSRPLND
LVSQKTTQLSDITSRFNSAIEALNRFIQKYDSVMQRLLDDTSGK

>199-90
MIRAYEQNPQHFIEDLEKVRVEQLTGHGSSVLEELVQLVKDKNIDISIKYDPRKDSEVFANRVITDDIELFKKILAYFLPEDAILKGGHYDNQL
QNGIKRVKEFLESSPNTQWELRAFMAVIHFSLTADRIDDDILKVIVDSMNHHGDARSKLREELAELTAELKIYSVIQAEINKHLSSSGTINIHD
ESINLMDKNLYGYTDDDIFKASAEYKILEKMPQTTIQVDGSEKKIVSIKDFLGSENKRTGALGNLKNSYSYNKDNNELSHFATTCSDKSRPLND
LVSQKTTQLSDITSRFNSAIEALNRFIQKYDSVMQRLLDDTSGK

>99-91
MIRAYEQNPQHFIEDLEKVRVEQLTGHGSSVLEELVQLVKDKNIDISIKYDPRKDSEVFANRVITDDIELFKKILAYFLPEDAILKGGHYDNQL
QNGIKRVKEFLESSPNTQWELRAFMAVIHFSLTADRIDDDILKVIVDSMNHHGDARSKLREELAELTAELKIYSVIQAEINKHLSSSGTINIHD
ESINLMDKNLYGYTDDDIFKASAEYKILEKMPQTTIQVDGSEKKIVSIKDFLGSENKRTGALGNLKNSYSYNKDNNELSHFATTCSDKSRPLND
LVSQKTTQLSDITSRFNSAIEALNRFIQKYDSVMQRLLDDTSGK

>CO92
MIRAYEQNPQHFIEDLEKVRVEQLTGHGSSVLEELVQLVKDKNIDISIKYDPRKDSEVFANRVITDDIELLKKILAYFLPEDAILKGGHYDNQL
QNGIKRVKEFLESSPNTQWELRAFMAVMHFSLTADRIDDDILKVIVDSMNHHGDARSKLREELAELTAELKIYSVIQAEINKHLSSSGTINIHD
KSINLMDKNLYGYTDEEIFKASAEYKILEKMPQTTIQVDGSEKKIVSIKDFLGSENKRTGALGNLKNSYSYNKDNNELSHFATTCSDKSRPLND
LVSQKTTQLSDITSRFNSAIEALNRFIQKYDSVMQRLLDDTSGK

>195/P
MIRAYEQNPQHFIEDLEKVRVEQLTGHGSSVLEELVQLVKDKNIDISIKYDPRKDSEVFANRVITDDIELLKKILAYFLPEDAILKGGHYDNQL
QNGIKRVKEFLESSPNTQWELRAFMAVMHFSLTADRIDDDILKVIVDSMNHHGDARSKLREELAELTAELKIYSVIQAEINKHLSSSGTINIHD
KSINLMDKNLYGYTDEEIFKASAEYKILEKMPQTTIQVDGSEKKIVSIKDFLGSENKRTGALGNLKNSYSYNKDNNELSHFATTCSDKSRPLND
LVSQKTTQLSDITSRFNSAIEALNRFIQKYDSVMQRLLDDTSGK

GROUP D

>AH
MIRAYEQNPQHFIEDLEKVRVEQLTGHGSSVLEELVQLVKDKNIDISIKYDPRKDSEVFANRVITDDIELLKKILAYFLPEDAILKGGHYDNQL
QNGIKRVKEFLESSPNTQWELRAFMAVMHFSLTADRIDDDILKVIVDSMNHHGDARSKLREELAELTAELKIYSVIQAEINKHLSSGGTINIHD
KSINLMDKNLYGYTDEEIFKASAEYKILEKMPQTTIQEGETEKKIVSIKNFLESEKKRTGALGNLKDSYSYNKDNNELSHFATTCSDKSRPLND
LVSQKTTQLSDITSRFNSAIEALNRFIQKYDSVMQRLLDDTSGK

>1432-94
MIRAYEQNPQHFIEDLEKVRVEQLTGHGSSVLEELVQLVKDKNIDISIKYDPRKDSEVFANRVITDDIELLKKILAYFLPEDAILKGGHYDNQL
QNGIKRVKEFLESSPNTQWELRAFMAVIHFSLTADRIDDDILKVIVDSMNHHGDARSKLREELAELTAELKIYSVIQAEINKHLSSGGTINIHD
KSINLMDKILYGYTDEEIFKASAEYKILEKMTTIQEGETEKKIVSIKNFLESEKKRTGALGNLKDSYSYNKDNNELSHFATTCSDKSRPLNDLV
SQKTTQLSDITSRFNSAIEALNRFIQKYDSVMQRLLDDTSGK

>297-89
MIRAYEQNPQHFIEDLEKVRVEQLTGHGSSVLEELVQLVKDKNIDISIKYDPRKDSEVFANRVITDDIELLKKILAYFLPEDAILKGGHYDNQL
QNGIKRVKEFLESSPNTQWELRAFMAVIHFSLTADRIDDDILKVIVDSMNHHGDARSKLREELAELTAELKIYSVIQAEINKHLSSGGTINIHD
KSINLMDKNLYGYTDEEIFKASAEYKILEKMTTIQEGETEKKIVSIKNFLESEKKRTGALGNLKDSYSYNKDNNELSHFATTCSDKSRPLNDLV
SQKTTQLSDITSRFNSAIEALNRFIQKYDSVMQRLLDDTSGK

>300-89
MIRAYEQNPQHFIEDLEKVRVEQLTGHGSSVLEELVQLVKDKNIDISIKYDPRKDSEVFANRVITDDIELLKKILAYFLPEDAILKGGHYDNQL
QNGIKRVKEFLESSPNTQWELRAFMAVIHFSLTADRIDDDILKVIVDSMNHHGDARSKLREELAELTAELKIYSVIQAEINKHLSSGGTINIHD
KSINLMDKNLYGYTDEEIFKASAEYKILEKMTTIQEGETEKKIVSIKNFLESEKKRTGALGNLKDSYSYNKDNNELSHFATTCSDKSRPLNDLV
SQKTTQLSDITSRFNSAIEALNRFIQKYDSVMQRLLDDTSGK

>165-89
MIRAYEQNPQHFIEDLEKVRVEQLTGHGSSVLEELVQLVKDKNIDISIKYDPRKDSEVFANRVITDDIELLKKILAYFLPEDAILKGGHYDNQL
QNGIKRVKEFLESSPNTQWELRAFMAVIHFSLTADRIDDDILKVIVDSMNHHGDARSKLREELAELTAELKIYSVIQAEINKHLSSGGTINIHD
KSINLMDKNLYGYTDEEIFKASAEYKILEKMTTIQEGETEKKIVSIKNFLESEKKRTGALGNLKDSYSYNKDNNELSHFATTCSDKSRPLNDLV
SQKTTQLSDITSRFNSAIEALNRFIQKYDSVMQRLLDDTSGK

>2783
MIRAYEQNPQHFIEDLEKVRVEQLTGHGSSVLEELVQLVKDKNIDISIKYDPRKDSEVFANRVITDDIELLKKILAYFLPEDAILKGGHYDNQL
QNGIKRVKEFLESSPNTQWELRAFMAVIHFSLTADRIDDDILKVIVDSMNHHGDARSKLREELAELTAELKIYSVIQAEINKHLSSGGTINIHD
KSINLMDKNLYGYTDEEIFKASAEYKILEKMPQTTIQEGETEKKIVSIKNFLESEKKRTGALGNLKDSYSYNKDNNELSHFATTCSDKSRPLND
LVSQKTTQLSDITSRFNSAIEALNRFIQKYDSVMQRLLDDTSGK

>2790
MIRAYEQNPQHFIEDLEKVRVEQLTGHGSSVLEELVQLVKDKNIDISIKYDPRKDSEVFANRVITDDIELLKKILAYFLPEDAILKGGHYDNQL
QNGIKRVKEFLESSPNTQWELRAFMAVIHFSLTADRIDDDILKVIVDSMNHHGDARSKLREELAELTAELKIYSVIQAEINKHLSSGGTINIHD

KSINLMDKNLYGYTDEEIFKASAEYKILEKMPQTTIQEGETEKKIVSIKNFLESEKKRTGALGNLKDSYSYNKDNNELSHFATTCSDKSRPLND
LVSQKTTQLSDITSRFNSAIEALNRFIQKYDSVMQRLLDDTSGK

>2853
MIRAYEQNPQHFIEDLEKVRVEQLTGHGSSVLEELVQLVKDKNIDISIKYDPRKDSEVFANRVITDDIELLKKILAYFLPEDAILKGGHYDNQL
QNGIKRVKEFLESSPNTQWELRAFMAVIHFSLTADRIDDDILKVIVDSMNHHGDARSKLREELAELTAELKIYSVIQAEINKHLSSGGTINIHD
KSINLMDKNLYGYTDEEIFKASAEYKILEKMPQTTIQEGETEKKIVSIKNFLESEKKRTGALGNLKDSYSYNKDNNELSHFATTCSDKSRPLND
LVSQKTTQLSDITSRFNSAIEALNRFIQKYDSVMQRLLDDTSGK

>WE27-93
MIRAYEQNPQHFIEDLEKVRVEQLTGHGSSVLEELVQLVKDKNIDISIKYDPRKDSEVFANRVITDDIELLKKILAYFLPEDAILKGGHYDNQL
QNGIKRVKEFLESSPNTQWELRAFMAVIHFSLTADRIDDDILKVIVDSMNHHGDARSKLREELAELTAELKIYSVIQAEINKHLSSGGTINIHD
KSINLMDKNLYGYTDEEIFKASAEYKILEKMPQTTIQEGETEKKIVSIKNFLESEKKRTGALGNLKDSYSYNKDNNELSHFATTCSDKSRPLND
LVSQKTTQLSDITSRFNSAIEALNRFIQKYDSVMQRLLDDTSGK

>YPTIV
MIRAYEQNPQHFIEDLEKVRVEQLTGHGSSVLEELVQLVKDKNIDISIKYDPRKDSEVFANRVITDDIELLKKILAYFLPEDAILKGGHYDNQL
QNGIKRVKEFLESSPNTQWELRAFMAVIHFSLTADRIDDDILKVIVDSMNHHGDARSKLREELAELTAELKIYSVIQAEINKHLSSGGTINIHD
KSINLMDKNLYGYTDEEIFKASAEYKILEKMPQTTIQEGETEKKIVSIKNFLESEKKRTGALGNLKDSYSYNKDNNELSHFATTCSDKSRPLND
LVSQKTTQLSDITSRFNSAIEALNRFIQKYDSVMQRLLDDTSGK

>2953
MIRAYEQNPQHFIEDLEKVRVEQLTGHGSSVLEELVQLVKDKNIDISIKYDPRKDSEVFANRVITDDIELLKKILAYFLPEDAILKGGHYDNQL
QNGIKRVKEFLESSPNTQWELRAFMAVIHFSLTADRIDDDILKVIVDSMNHHGDARSKLREELAELTAELKIYSVIQAEINKHLSSGGTINIHD
KSINLMDKNLYGYTDEEIFKASAEYKILEKMPQTTIQEGETEKKIVSIKNFLESEKKRTGALGNLKDSYSYNKDNNELSHFATTCSDKSRPLND
LVSQKTTQLSDITSRFNSAIEALNRFIQKYDSVMQRLLDDTSGK

>PB1/+
MIRAYEQNPQHFIEDLEKVRVEQLTGHGSSVLEELVQLVKDKNIDISIKYDPRKDSEVFANRVITDDIELLKKILAYFLPEDAILKGGHYDNQL
QNGIKRVKEFLESSPNTQWELRAFMAVIHFSLTADRIDDDILKVIVDSMNHHGDARSKLREELAELTAELKIYSVIQAEINKHLSSGGTINIHD
KSINLMDKNLYGYTDEEIFKASAEYKILEKMPQTTIQEGETEKKIVSIKNFLESEKKRTGALGNLKDSYSYNKDNNELSHFATTCSDKSRPLND
LVSQKTTQLSDITSRFNSAIEALNRFIQKYDSVMQRLLDDTSGK

>2889
MIRAYEQNPQHFIEDLEKVRVEQLTGHGSSVLEELVQLVKDKNIDISIKYDPRKDSEVFANRVITDDIELLKKILAYFLPEDAILKGGHYDNQL
QNGIKRVKEFLESSPNTQWELRAFMAVIHFSLTADRIDDDILKVIVDSMNHHGDARSKLREELAELTAELKIYSVIQAEINKHLSSGGTINIHD
KSINLMDKNLYGYTDEEIFKASAEYKILEKMPQTTIQEGETEKKIVSIKNFLESEKKRTGALGNLKDSYSYNKDNNELSHFATTCSDKSRPLND
LVSQKTTQLSDITSRFNSAIEALNRFIQKYDSVMQRLLDDTSGK

>2666
MIRAYEQNPQHFIEDLEKVRVEQLTGHGSSVLEELVQLVKDKNIDISIKYDPRKDSEVFANRVITDDIELLKKILAYFLPEDAILKGGHYDNQL
QNGIKRVKEFLESSPNTQWELRAFMAVIHFSLTADRIDDDILKVIVDSMNHHGDARSKLREELAELTAELKIYSVIQAEINKHLSSGGTINIHD
KSINLMDKNLYGYTDEEIFKASAEYKILEKMPQTTIQEGETEKKIVSIKNFLESEKKRTGALGNLKDSYSYNKDNNELSHFATTCSDKSRPLND
LVSQKTTQLSDITSRFNSAIEALNRFIQKYDSVMQRLLDDTSGK

>YPIII
MIRAYEQNPQHFIEDLEKVRVEQLTGHGSSVLEELVQLVKDKNIDISIKYDPRKDSEVFANRVITDDIELLKKILAYFLPEDAILKGGHYDNQL
QNGIKRVKEFLESSPNTQWELRAFMAVIHFSLTADRIDDDILKVIVDSMNHHGDARSKLREELAELTAELKIYSVIQAEINKHLSSGGTINIHD
KSINLMDKNLYGYTDEEIFKASAEYKILEKMPQTTIQEGETEKKIVSIKNFLESEKKRTGALGNLKDSYSYNKDNNELSHFATTCSDKSRPLND
LVSQKTTQLSDITSRFNSAIEALNRFIQKYDSVMQRLLDDTSGK

>32977
MIRAYEQNPQHFIEDLEKVRVEQLTGHGSSVLEELVQLVKDKNIDISIKYDPRKDSEVFANRVITDDIELLKKILAYFLPEDAILKGGHYDNQL
QNGIKRVKEFLESSPNTQWELRAFMAVIHFSLTADRIDDDILKVIVDSMNHHGDARSKLREELAELTAELKIYSVIQAEINKHLSSGGTINIHD
KSINLMDKNLYGYTDEEIFKASAEYKILEKMPQTTIQEGETEKKIVSIKNFLESEKKRTGALGNLKDSYSYNKDNNELSHFATTCSDKSRPLND
LVSQKTTQLSDITSRFNSAIEALNRFIQKYDSVMQRLLDDTSGK

>32984
MIRAYEQNPQHFIEDLEKVRVEQLTGHGSSVLEELVQLVKDKNIDISIKYDPRKDSEVFANRVITDDIELLKKILAYFLPEDAILKGGHYDNQL
QNGIKRVKEFLESSPNTQWELRAFMAVIHFSLTADRIDDDILKVIVDSMNHHGDARSKLREELAELTAELKIYSVIQAEINKHLSSGGTINIHD
KSINLMDKNLYGYTDEEIFKASAEYKILEKMPQTTIQEGETEKKIVSIKNFLESEKKRTGALGNLKDSYSYNKDNNELSHFATTCSDKSRPLND
LVSQKTTQLSDITSRFNSAIEALNRFIQKYDSVMQRLLDDTSGK

>32861

MIRAYEQNPQHFIEDLEKVRVEQLTGHGSSVLEELVQLVKDKNIDISIKYDPRKDSEVFANRVITDDIELLKKILAYFLPEDAILKGGHYDNQL
QNGIKRVKEFLESSPNTQWELRAFMAVIHFSLTADRIDDDILKVIVDSMNHHGDARSKLREELAELTAELKIYSVIQAEINKHLSSGGTINIHD
KSINLMDKNLYGYTDEEIFKASAEYKILEKMPQTTIQEGETEKKIVSIKNFLESEKKRTGALGNLKDSYSYNKDNNELSHFATTCSDKSRPLND
LVSQKTTQLSDITSRFNSAIEALNRFIQKYDSVMQRLLDDTSGK

>KM
MIRAYEQNPQHFIEDLEKVRVEQLTGHGSSVLEELVQLVKDKNIDISIKYDPRKDSEVFANRVITDDIELLKKILAYFLPEDAILKGGHYDNQL
QNGIKRVKEFLESSPNTQWELRAFMAVIHFSLTADRIDDDILKVIVDSMNHHGDARSKLREELAELTAELKIYSVIQAEINKHLSSGGTINIHD
KSINLMDKNLYGYTDEEIFKASAEYKILEKMPQTTIQEGETEKKIVSIKNFLESEKKRTGALGNLKDSYSYNKDNNELSHFATTCSDKSRPLND
LVSQKTTQLSDITSRFNSAIEALNRFIQKYDSVMQRLLDDTSGK

>ST
MIRAYEQNPQHFIEDLEKVRVEQLTGHGSSVLEELVQLVKDKNIDISIKYDPRKDSEVFANRVITDDIELLKKILAYFLPEDAILKGGHYDNQL
QNGIKRVKEFLESSPNTQWELRAFMAVIHFSLTADRIDDDILKVIVDSMNHHGDARSKLREELAELTAELKIYSVIQAEINKHLSSGGTINIHD
KSINLMDKNLYGYTDEEIFKASAEYKILEKMPQTTIQEGETEKKIVSIKNFLESEKKRTGALGNLKDSYSYNKDNNELSHFATTCSDKSRPLND
LVSQKTTQLSDITSRFNSAIEALNRFIQKYDSVMQRLLDDTSGK

>NT
MIRAYEQNPQHFIEDLEKVRVEQLTGHGSSVLEELVQLVKDKNIDISIKYDPRKDSEVFANRVITDDIELLKKILAYFLPEDAILKGGHYDNQL
QNGIKRVKEFLESSPNTQWELRAFMAVMHFSLTADRIDDDILKVIVDSMNHHGDARSKLREELAELTAELKIYSVIQAEINKHLSSSGTINIHD
KSINLMDKNLYGYTDEAIFKASAEYKILEKMPQTNTTQVDGSEKKIVSIKDFLGSEKKRTGALGNLKDSYSYNKDNNELSHFATTCSDKSRPLN
DLVSQKTTQLSDITSRFNSAIEALNRFIQKYDSVMQRLLDDTSGK

>3255-93
MIRAYEQNPQHFIEDLEKVRVEQLTGHGSSVLEELVQLVKDKNIDISIKYDPRKDSEVFANRVITDDIELLKKILAYFLPEDAILKGGHYDNQL
QNGIKRVKEFLESSPNTQWELRAFMAVIHFSLTADRIDDDILKVIVDSMNHHGDARSKLREELAELTAELKIYSVIQAEINKHLSSGGTINIHD
KSINLMDKNLYGYTDEEIFKASAEYKILEKMPQTTIQEGETEKKIVSIKNFLESEKKRTGALGNLKDSYSYNKDNNELSHFATTCSDKSRPLND
LVSQKTTQLSDITSRFNSAIEALNRFIQKYDSVMQRLLDDTSGK
